# Synthesis of hypercrosslinked polymers using coconut oil as a renewable, bio-based solvent

**DOI:** 10.1039/d5gc03906a

**Published:** 2025-10-21

**Authors:** Paul Schweng, Anastasiia Naryshkina, Alexander Blocher, Robert T. Woodward

**Affiliations:** a Institute of Materials Chemistry and Research, Faculty of Chemistry, University of Vienna Währinger Straße 42 1090 Vienna Austria robert.woodward@univie.ac.at; b Vienna Doctoral School in Chemistry, University of Vienna Währinger Straße 42 1090 Vienna Austria

## Abstract

We report a sustainable approach to the synthesis of hypercrosslinked polymers by replacing conventional chlorinated solvents with renewable bio-based oils. Hypercrosslinked polymers are widely used porous materials in both academic and industrial settings, yet their synthesis remains heavily reliant on chlorinated solvents subject to growing regulatory restrictions due to their environmental and health hazards. Here, we identify coconut oil as an effective alternative solvent for the synthesis of high surface area hypercrosslinked polymers (>400 m^2^ g^−1^) with excellent thermal and chemical stability. We explore the role of fatty acid composition in the oil, finding that saturated acid content correlates to network porosity. By demonstrating that benign, renewable substrates can replace hazardous solvents, this work provides a timely and practical step toward greener polymer production methods.

## Abstract 2

We report a sustainable approach to the synthesis of hypercrosslinked polymers by replacing conventional chlorinated solvents with renewable bio-based oils.

## Graphical abstract

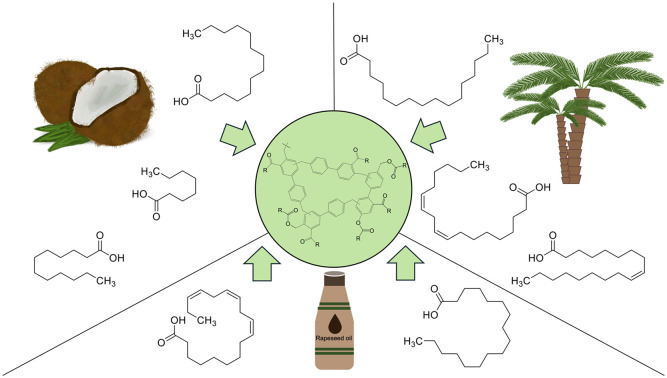

**Green foundation**

1. In this work, hazardous chlorinated solvents were replaced with bio-based oils for the synthesis of hypercrosslinked polymers. Traditional chlorinated solvents like dichloromethane or dichloroethane pose significant environmental and health risks due to their persistence, volatility, and toxicity. By demonstrating that naturally derived oils can effectively replace these solvents while producing highly porous networks, this research offers a safer, more sustainable route for porous polymer production.

2. Successful replacement of traditional chlorinated solvents with coconut oil led to the formation of highly porous materials with surface areas reaching >400 m^2^ g^−1^.

3. In the future, we will seek to utilise waste cooking oils, triglycerides, or alternative bio-based oils both to improve network porosity and further demonstrate the utility of bio-solvents. We will also seek to reduce the amount of Lewis acid catalyst required for the polymerisation upon the discovery of more suitable bio-solvents. Finally, we will investigate the application of our process to Davankov resins and the scale-up of our process to seek alternative, greener routes to commercial porous resins, such as ion exchange resins.

## Introduction

Hypercrosslinked polymers (HCPs), originally developed by Davankov and co-workers,^1^ have become foundational materials in a wide array of applications ranging from ion exchange and chromatographic separation to gas capture, catalysis, and pollutant adsorption.^2,3^ Their appeal lies in their exceptional surface area, chemical resilience, and the tuneability of their pore architecture.^4^ However, the synthesis of HCPs has remained reliant on hazardous chlorinated solvents such as dichloromethane (DCM) or 1,2-dichloroethane (DCE),^5,6^ which deeply embed them in commercial-scale production and academic methods. With the global market for HCPs estimated to reach >$4 billion by 2034,^7^ urgent innovation is required to avoid the generation of significant hazardous halogenated solvent waste.

Chlorinated solvents are commonly used across industries for their strong solvating capabilities, despite growing interest in green alternatives.^8^ However, large-scale industrial use of chlorinated solvents can pose serious health risks to workers, while also contributing to long-term environmental contamination of air, water, and soil.^9,10^ Occupational exposure to volatile chlorinated solvents, primarily through inhalation in poorly ventilated environments, is particularly concerning. Prolonged or high-concentration contact has been linked to a range of health issues, including neurotoxicity, liver and kidney damage, reproductive effects, and increased cancer risk.^11,12^ Chlorinated solvents have also been associated with detrimental developmental effects in unborn children, including neural tube and cardiac effects, particularly among mothers residing near industrial emissions.^13^

From an environmental perspective, chlorinated solvents are resistant to biodegradation, due to the strength of the carbon–chlorine bond and the limited capacity of microbial systems to break them down.^14^ This renders these solvents persistent and widespread environmental contaminants,^10^ tending to accumulate in ecosystems, particularly in groundwater, where they pose potential long-term risks due to bioaccumulation. In water, halogenated solvents can undergo dehalogenation reactions, leading to their breakdown, however, the resulting byproducts are often more toxic than the original compounds.^15^

Due to the significant risks that chlorinated solvents pose to human health and the environment, the European Parliament has implemented strict regulatory control on their use and disposal. These measures are designed not only to manage exposure but also to encourage the adoption of safer alternatives.^16^ Despite these efforts, substitution has often led to the switching to other hazardous solvents, rather than benign alternatives.^17^ The substitution of chlorinated solvents with bio-based solvent would reduce toxic waste generation and improve the environmental compatibility of HCP synthesis, enhancing prospects for sustainable industrial implementation. One approach for more sustainable HCP synthesis is the use of deep eutectic solvents as green alternatives, demonstrating a surface area reduction of just 22% compared to equivalents made in chlorinated solvents.^18^ However, such examples remain scarce. As regulatory pressure intensifies, there is an urgent need to identify greener, safer alternatives for HCP synthesis that do not significantly compromise material performance.

Here, we investigate the use of coconut oil, a biodegradable and non-toxic alternative sourced from nature, as a green reaction medium for HCP synthesis. We optimise reagent ratios to maximise HCP porous properties and explore the use of different vegetable oils to better understand how fatty acid composition influences network formation. By replacing harmful chlorinated solvents with a renewable feedstock like coconut oil, our work contributes to the development of greener synthetic pathways for HCP materials.

## Results and discussion

**Fig. 1:**
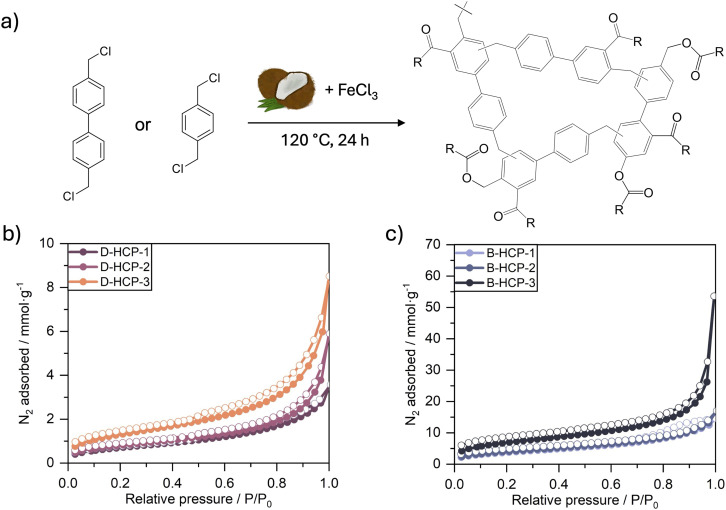
(a) Representative reaction scheme for the synthesis of HCPs using coconut oil solvent. (b) N_2_ adsorption–desorption isotherms of D-HCPs, and (c) B-HCPs.

A series of HCPs was synthesised by dissolving either 4,4′-bis(chloromethyl)-1,1′-biphenyl (BCMBP) or α,α′-dichloro-*p*-xylene (DCX) in coconut oil, which served as a bio-based alternative to conventional chlorinated solvents, such as DCE or DCM. Polymerisation was initiated *via* the addition of iron(iii) chloride (FeCl_3_) as a Lewis acid catalyst, and the reactions were conducted at 120 °C for 24 h (Fig. 1a). Three monomer-to-catalyst molar ratios (1 : 1, 1 : 2, and 1 : 3) were explored to assess their influence on the porosity of the resulting networks. Network yields varied between 125% and 200%, depending on the reagent feed (Table 1), and were calculated using eqn (S1) (SI), assuming the full conversion of chloromethyl groups into methylene crosslinks. Samples were washed thoroughly with heptane and methanol to remove residual oil, therefore, excess yields are attributed to fatty acid incorporation into the polymer network and some incomplete chloromethyl groups condensation. Both effects are investigated further below. The polymers were designated B-HCP-*n* or D-HCP-*n*, with B or D corresponding to the use of BCMBP or DCX, respectively, and *n* representing the ratio of catalyst added with respect to monomer, *e.g.* B-HCP-3 is an HCP produced from BCMBP with a monomer-to-catalyst ratio of 1 : 3. A detailed synthetic procedure is provided in the SI (Tables S1 and S2).

**Table 1:** Outline of HCP composition and yield, as well as their porous properties, including BET surface area, micropore volume, *V*_micro_, and total pore volume, *V*_tot_

Network	Monomer	Monomer to catalyst molar ratio	Yield (%)	Surface area (m^2^ g^−1^)	*V*_micro_ (cm^3^ g^−1^)	*V*_tot_ (cm^3^ g^−1^)
B-HCP-1	BCMBP	1 : 1	125	315 ± 15	<0.01	0.50 ± 0.09
B-HCP-2	BCMBP	1 : 2	145	359 ± 87	<0.01	0.51 ± 0.14
B-HCP-3	BCMBP	1 : 3	150	447 ± 94	0.015 ± 0.003	0.66 ± 0.41
D-HCP-1	DCX	1 : 1	173	54 ± 2	<0.01	0.09 ± 0.01
D-HCP-2	DCX	1 : 2	193	58 ± 7	<0.01	0.09 ± 0.03
D-HCP-3	DCX	1 : 3	200	96 ± 9	<0.01	0.16 ± 0.03

Nitrogen adsorption–desorption isotherms were measured at −196 °C to determine the porous properties of the HCPs produced using coconut oil. The isotherms exhibit both type I and type IVa features according to IUPAC classifications,^19^ indicating some microporosity *via* uptake at low relative pressures in B-HCP-3 and mesoporosity due to capillary condensation as shown by hysteresis in all isotherms (Fig. 1b and c). Gas sorption measurements confirmed the formation of porous networks, with surface areas varying from 50 to 440 m^2^ g^−1^ depending on monomer-to-catalyst ratio (Table 1). B-HCP-3 exhibited the highest surface area, indicating that increased catalyst loading led to an enhanced crosslinking density (Table 1). Further increases in the monomer-to-catalyst ratio were not pursued due to the limited solubility of the monomer and catalyst in coconut oil.

Thermal stability of the HCPs was assessed using thermogravimetric analysis (TGA) in air. Samples were heated to 700 °C at a rate of 10 °C min^−1^. Minimal weight loss was observed for all samples up to 300 °C (Fig. S1), after which degradation began. All polymers were completely degraded by ∼550 °C, as indicated by complete mass loss.

**Fig. 2:**
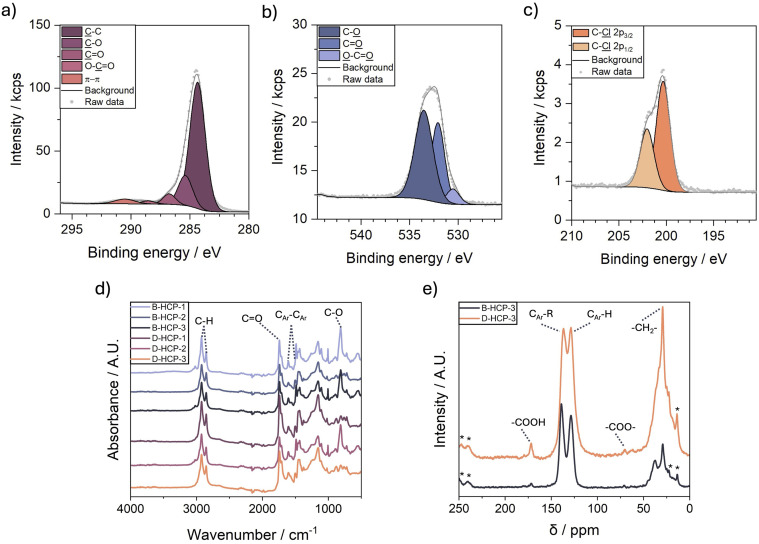
(a) B-HCP-3 high-resolution C 1s XPS spectrum, (b) B-HCP-3 high-resolution O 1s XPS spectrum, (c) B-HCP-3 high-resolution Cl 2p spectrum, (d) FTIR spectra of all B-HCPs and D-HCPs, (e) ^13^C cross-polarisation/magic angle spinning solid-state NMR of B-HCP-3 and D-HCP-3. * Represents spinning side bands.

X-ray photoelectron spectroscopy (XPS) analysis of B-HCP-3 revealed features corresponding to oxygen-containing moieties within the polymer matrix. The high-resolution C 1s spectrum (Fig. 2a) displayed a dominant peak at 284.8 eV, assigned to C–C bonding, encompassing both aromatic sp^2^ and aliphatic sp^3^ carbon. A peak at 285.4 eV corresponds to C–O groups, a signal at 286.8 eV was attributed to C=O functionalities, while an additional peak at 288.6 eV was assigned to O–C=O moieties, indicative of ester or carboxylic acid groups, possibly formed from oil-derived components. Coconut oil is composed of triglycerides comprising a glycerol backbone and three, predominantly saturated, fatty acids, such as lauric, myristic, and palmitic acids, which are rich in oxygen-containing esters and carboxylic acids in their free form.^20^ A low-intensity, broad π–π* shakeup was observed at 290.5 eV, confirming the presence of aromatic structures. The O 1s spectrum (Fig. 2b) of B-HCP-3 displayed peaks at 530.6 eV, 532.1 eV, and 533.6 eV assigned to O–C=O, C=O, and C–O environments, respectively, corroborating the oxygen-containing moieties observed in the C 1s spectrum. The high-resolution Cl 2p spectrum (Fig. 2c) exhibited weak signals at 200.4 eV and 202.1 eV for Cl 2p_3/2_ and Cl 2p_1/2_, suggesting a high degree of crosslinking with only some residual organic C–Cl bonds. The overall surface compositions determined by XPS are provided in Table S3. The XPS results strongly suggest that fatty acid species from the coconut oil were integrated into the polymer network *via* side reactions such as electrophilic substitution or esterification. Under the Lewis acidic conditions provided by FeCl_3_, carboxylic acid groups present in the oil can react with monomeric carbocation intermediates, resulting in ester linkage formation. The incorporation of R–C=O groups into the polymer structure may occur *via* electrophilic aromatic substitution, consistent with an acylation-type mechanism.^21,22^ The O–C=O moieties may also be attributed to carboxylic acids incorporated into the networks *via* addition across the double bonds of unsaturated fatty acids.^23^ The proposed side reactions between the polymer network and fatty acids are depicted in Fig. S2 and likely result in the high apparent yields observed when using coconut oil as solvent.

Elemental analysis (CHNS-O, EA) was used to determine the bulk chemical composition of the HCPs (Table S4). Samples synthesised with coconut oil displayed significantly higher oxygen contents than those prepared *via* conventional DCE based routes,^24,25^ in line with the proposed incorporation of fatty acids into the networks. Both EA and XPS revealed consistent compositional trends, with slight deviations in C and O contents due to XPS's inability to detect hydrogen and EA's inability to measure chlorine.

Fourier-transform infrared spectroscopy (FTIR) confirmed the presence of carbonyl functionalities introduced through the incorporation of coconut oil species (Fig. 2d). A sharp peak at 1701 cm^−1^ indicates the presence of C=O stretching, consistent with carboxylic acid or ester functionalities. Bands observed at 1400, and 1600 cm^−1^ are assigned to aromatic ring skeletal vibrations, while signals at 2900 cm^−1^ and 3020 cm^−1^ are attributed to aliphatic and aromatic –CH stretching, respectively. The signal at 1460 cm^−1^ is characteristic of –CH_2_ groups, derived from both crosslinks and incorporated fatty acids.

We collected ^13^C cross-polarisation/magic angle spinning solid-state NMR (CP/MAS ssNMR) spectra to further confirm the incorporation of fatty acids into the polymer network (Fig. 2e and S3). Characteristic signals for HCPs were observed at 139, 129, and 42 ppm, corresponding to substituted aromatic carbon (C_Ar_–R), aromatic carbon (C_Ar_–H), and residual chloromethyl groups, respectively.^24,25^ Additional signals, which are typically absent in HCPs synthesised using 1,2-dichloroethane, appeared at 172 and 64 ppm, and are assigned to carboxylic acid and benzylic ester groups, respectively. Furthermore, the carbon chain methylene groups of the incorporated fatty acids led to a significantly increased intensity of the signal at 30 ppm. Interestingly, resonances associated with oxygenated species were weak, and no clear signals for ketones were detected, despite their presence in XPS and FTIR. This discrepancy is most likely due to the use of cross-polarisation, which can weaken or even suppress carbons lacking directly bonded protons.^26^

The synthesis of HCPs using coconut oil was compared to a conventional approach using the complete environmental impact factor (*E* factor, cEF).^27^ The ideal *E* factor is zero, and a higher *E* factor means more waste generation. The cEF for B-HCP-3 was 249, while an equivalent polymer synthesised in 1,2-DCE had a cEF of 365. The reduced cEF of the B-HCP-3 is due to the incorporation of fatty acids, increasing the product mass, and the higher density of 1,2-dichloroethane (1.25 g cm^−3^) compared to coconut oil (0.92 g cm^−3^). The relatively high *E* factor values are due to the use of excess methanol as wash solvent; however, it should be noted that we recycle our wash solvent in a Soxhlet extractor. Considering solvent recycling, our cEFs fall to 31 and 47 for B-HCP-3 and its 1,2-DCE equivalent, respectively. The cEF equation and masses used for the calculations are provided in the SI (eqn (S2), Tables S5 and S6).

We attempted coconut oil recovery from a scaled-up reaction employing 25 mL of the bio-based solvent (Experimental details are provided in the SI). After extraction with heptane and water, 14 mL (56%) of the coconut oil was recovered. The recovered oil exhibited a slightly orange color (Fig. S4), attributed to trace iron residues. The discrepancies between the recovered and initial amounts are likely due to handling losses during washing and transfer, as well as some incorporation into the polymer network. We acknowledge that this approach has limitations, but it serves to demonstrate the ability to recover the system solvent post-synthesis. It should also be noted that the heptane used for extraction was fully recoverable.

Following the successful application of coconut oil as a renewable solvent for HCP synthesis, palm oil and rapeseed oil were explored under identical reaction conditions, including variation in the monomer-to-catalyst molar ratios. These oils were selected due to their widespread availability and fatty acid compositions. In contrast to coconut oil, which has a fatty acid composition comprising ∼93% saturated fatty acids, palm oil and rapeseed oil contain ∼46% and 8% saturated fatty acids,^28,29^ respectively. The resulting polymers were named according to the previously established convention, with the suffixes “PO” and “RO” denoting the use of palm and rapeseed oil, respectively. Apparent product yields reached up to 322% for palm oil and 863% for rapeseed oil (Table S2), attributed to the enhanced incorporation of unsaturated fatty acids into the polymer network relative to coconut oil, consistent with the increased oxygen content detected in EA (Table S5).

Both palm and rapeseed oil-derived polymers exhibited negligible surface area and nitrogen uptake (Table S7), indicating the absence of porosity. Coupled with their high yields, the lack of significant porosity implies that the degree of saturation in the solvent's fatty acid components play a critical role in network formation.

Further characterisation by XPS confirmed incorporation of oil-derived moieties (Fig. S5–S7). XPS analysis of B-HCP-3-PO and B-HCP-3-RO confirmed the presence of oxygen-containing functionalities associated with oil incorporation, as discussed above. Notably, the intensity of the O–C=O signal increased progressively across B-HCP-3, B-HCP-3-PO, and B-HCP-3-RO, indicating a higher degree of fatty acid incorporation from the palm oil and rapeseed oil, respectively. This trend was further supported by the O 1s spectra. The XPS results again suggest that the unsaturated fatty acids are more readily incorporated into the polymer matrix, leading to the higher network yields. High network yields may also be due to the retention of some residual adsorbed oil, *i.e.* not chemically bound.

To assess the impact of fatty acid saturation on polymer formation, control experiments were conducted using lauric acid (LA) or oleic acid (OA) as solvent. Lauric acid, a saturated fatty acid, is the primary component of coconut oil, whereas oleic acid, a monounsaturated fatty acid, predominates in rapeseed oil. HCPs were synthesised using either BCMBP or DCX with a fixed monomer-to-catalyst molar ratio of 1 : 3.^28,29^ The FTIR spectra of all networks are provided in Fig. S8.

Gas sorption analysis (Fig. S9, and Table S7) showed that polymers synthesised in lauric acid were porous (32 m^2^ g^−1^ for B-HCP-LA and 41 m^2^ g^−1^ D-HCP-LA) while those formed in oleic acid were non-porous. The surface area of B-HCP-LA was lower than that of equivalent networks produced in coconut oil, despite both systems containing a high proportion of saturated fatty acids. This discrepancy may be partly attributed to differences in solvent viscosity but could also reflect compositional or physical distinctions between pure lauric acid and the complex mixture of triglycerides in coconut oil. The multicomponent nature of coconut oil, comprising mostly saturated triglycerides with some minor unsaturated species, creates a more favourable environment for network expansion or pore formation during polymerisation. Nevertheless, these findings confirm that the presence of saturated fatty acids permit porous network formation, while unsaturated species like oleic acid may lead to further side reactions or disrupt crosslinking pathways during polymerisation, inhibiting porous network formation. Our findings underscore the importance of bio-based solvent composition for HCP synthesis, when seeking sustainable alternatives to chlorinated solvents.

## Conclusion

We demonstrate a more sustainable approach to hypercrosslinked polymer synthesis by replacing conventional chlorinated solvents with coconut oil to produce networks with surface areas of over 400 m^2^ g^−1^. Structural and compositional analysis confirmed partial incorporation of fatty acid species from the coconut oil into the polymer matrix *via* electrophilic substitution and esterification, influencing network porosity and resulting in high apparent yields >125%. The use of palm and rapeseed oils, both containing higher unsaturated fatty acid contents, resulted in significantly lower porosity and higher product yields, indicative of increased fatty acid incorporation *via* addition across the double bond. Control experiments with lauric and oleic acids as model solvents confirmed that saturated fatty acids are essential for pore development, likely by limiting their incorporation into the HCP structures compared to unsaturated equivalents. These findings underline the role of bio-based solvent composition in HCP synthesis. Beyond reducing reliance on hazardous chlorinated solvents, the use of abundant, low-cost bio-oils in an established Friedel–Crafts process underscores the scalability and industrial relevance of this greener route to porous hypercrosslinked polymers.

## Author contributions

A. B. conceived the idea. P. S. synthesised HCPs. P. S. and A. N. characterised HCPs. P. S and R. T. W. designed experiments. R. T. W. supervised this work. A. N. wrote the initial draft of the manuscript. A. N., P. S. and R. T. W. wrote and edited the manuscript, and all authors discussed the results and commented on the manuscript.

## Conflicts of interest

There are no conflicts of interest to declare.

## Supplementary Material

GC-027-D5GC03906A-s001

## Data Availability

Data for this article, including gas sorption and spectroscopic datasets (FTIR, XPS, ssNMR), thermogravimetric analyses, and data underlying the figures and tables in the text are available at Zenodo at https://doi.org/10.5281/zenodo.16572485. Supplementary information is available. See DOI: https://doi.org/10.1039/d5gc03906a.
