# Supplementary material for: Synthesis of hypercrosslinked polymers using coconut oil as a renewable, bio-based solvent
Source: Green Chem. 2025 Oct 21;27(46):14776–82. doi: 10.1039/d5gc03906a (PMC12593190; doi:10.1039/d5gc03906a)
Supplement: GC-027-D5GC03906A-s001 [file GC-027-D5GC03906A-s001.pdf]

Supplementary Information (SI) for Green Chemistry.
This journal is © The Royal Society of Chemistry 2025

Supporting Information

Synthesis of Hypercrosslinked Polymers Using Coconut Oil as Renewable, Bio-Based Solvent

Paul Schweng,[a,b]† Anastasiia Naryshkina,[a,]† Alexander Blocher,[a] Robert T. Woodward[a,]*

[a] Institute of Materials Chemistry and Research, Faculty of Chemistry, University of Vienna, Währinger Straße 42, 1090, Vienna, Austria

[b] Vienna Doctoral School in Chemistry, University of Vienna, Währinger Straße 42, 1090 Vienna, Austria

Email: robert.woodward@univie.ac.at

† These authors contributed equally to this work.

Keywords: hypercrosslinked polymers; green solvent; bio-based; porous organic polymer

Experimental

Materials

For the synthesis and purification process of hypercrosslinked polymers, 4,4'-bis(chloromethyl)-1,1'-biphenyl (BCMBP, 95%), iron (III) chloride (reagent grade, 97%), α,α'-dichloro-p-xylene (DCX, 98%), oleic acid (technical grade 90%), lauric acid (≥98%), heptane (HPLC grade, >99.9%) and methanol (HPLC grade, ≥99.8%) were purchased from Sigma Aldrich and used as received. Coconut oil, rapeseed oil and palm oil were purchased from a local supermarket.

Synthesis of hypercrosslinked polymers using bio-based oils as solvent

BCMBP (1.0 g, 4 mmol) or DCX (0.7 g, 4 mmol) were dissolved and stirred (10 min, ~350 rpm, 120 °C) in 5 mL of coconut oil (or palm oil or rapeseed oil). Iron(III) chloride was added to the heated solution to initiate polymerisation. A reflux condenser was attached, and the reaction was heated at 120 °C while stirring for 24 h. The resulting black-brown solid was washed with heptane in a Büchner funnel before being washed in a Soxhlet extractor with methanol, overnight. The sample was allowed to dry in the fume cupboard to remove excess methanol and then in the oven at 80 °C for 24 h. The dried polymer was subsequently ground gently with a pestle and mortar to obtain the final product as a fine powder. Precise reagent amounts used in the HCP syntheses are provided in Table S1. Product yields for all polymerisation reactions are provided in Table S2.

Synthesis of hypercrosslinked polymers using lauric acid or oleic acid as solvent

BCMBP (1.0 g, 4 mmol) or DCX (0.7 g, 4 mmol) were dissolved and stirred (10 min, ~350 rpm, 120 °C) in 5 mL of oleic acid or lauric acid. Iron(III) chloride was added to the heated solution to initiate polymerisation. A reflux condenser was attached, and the reaction was heated at 120 °C while stirring for 24 h. The resulting black-brown solid was washed with heptane in a Büchner funnel before being washed in a Soxhlet extractor with methanol, overnight. The sample was allowed to dry in the fume cupboard to remove excess methanol and then in the oven at 80 °C for 24 h. The dried polymer was subsequently ground gently with a pestle and mortar to obtain the final product as a fine powder. Precise reagent amounts used in the HCP syntheses are provided in Table S1. Product yields for all polymerisation reactions are provided in Table S2.

Oil recovery after synthesis

A scaled-up synthesis was performed using 25 mL of coconut oil as solvent. After quenching with water, the product was collected in a Büchner funnel and washed with ~25 mL each of water and n-heptane. The filtrate was transferred to a separatory funnel, and the heptane phase was washed twice more with water to remove residual $FeCl_3$. The organic layer was dried over $MgSO_4$, and heptane was removed using a rotary evaporator, yielding 12.9 g (14 mL) of coconut oil (Figure S4).

Table S1. Reagent quantities used in HCP Synthesis.

Sample	Monomer: catalyst (molar ratio)	Solvent	Monomer	Solvent (mL)	Catalyst (g)	Monomer (g)
B-HCP-1	1:1	coconut oil	BCMBP	5	0.646	1.004
B-HCP-2	1:2	coconut oil	BCMBP	5	1.299	1.006
B-HCP-3	1:3	coconut oil	BCMBP	5	1.945	1.004
D-HCP-1	1:1	coconut oil	DCX	5	0.649	0.700
D-HCP-2	1:2	coconut oil	DCX	5	1.302	0.701
D-HCP-3	1:3	coconut oil	DCX	5	1.951	0.701
B-HCP-1-PO	1:1	palm oil	BCMBP	5	0.646	1.003
B-HCP-2-PO	1:2	palm oil	BCMBP	5	1.302	1.006
B-HCP-3-PO	1:3	palm oil	BCMBP	5	1.949	1.005
D-HCP-1-PO	1:1	palm oil	DCX	5	0.649	0.700
D-HCP-2-PO	1:2	palm oil	DCX	5	1.294	0.699
D-HCP-3-PO	1:3	palm oil	DCX	5	1.956	0.701
B-HCP-1-RO	1:1	rapeseed oil	BCMBP	5	0.653	1.006
B-HCP-2-RO	1:2	rapeseed oil	BCMBP	5	1.304	1.005
B-HCP-3-RO	1:3	rapeseed oil	BCMBP	5	1.944	1.006
D-HCP-1-RO	1:1	rapeseed oil	DCX	5	0.651	0.699
D-HCP-2-RO	1:2	rapeseed oil	DCX	5	1.298	0.699
D-HCP-3-RO	1:3	rapeseed oil	DCX	5	1.045	0.701
B-HCP-LA	1:3	lauric acid	BCMBP	5	1.299	1.006
D-HCP-LA	1:3	lauric acid	DCX	5	1.294	0.700
B-HCP-OA	1:3	oleic acid	BCMBP	5	1.288	1.003
D-HCP-OA	1:3	oleic acid	DCX	5	1.308	0.701

Equation 1. Calculation of HCP yields. *m* represents mass in grams and *M* the molar mass in $g{\cdot}mol^{-1}$. BCMBP is the crosslinker 4,4'-bis(chloromethyl)-1,1'-biphenyl.

$$Yield(\%) = 100 \cdot \frac{m(product)}{m(BCMBP) - (2 \cdot M(HCl) \cdot \frac{m(BCMBP)}{M(BCMBP)}}$$

Table S2. HCP yields.

Sample name	Yield (g)	Yield (%)
B-HCP-1	0.888	125
B-HCP-2	0.708	173
B-HCP-3	1.036	145
D-HCP-1	0.791	193
D-HCP-2	1.069	150
D-HCP-3	0.819	200
B-HCP-1-PO	2.289	322
B-HCP-2-PO	0.194	47
B-HCP-3-PO	2.134	299
D-HCP-1-PO	0.413	101
D-HCP-2-PO	2.289	321
D-HCP-3-PO	1.129	276
B-HCP-1-RO	3.410	478
B-HCP-2-RO	3.520	863
B-HCP-3-RO	3.715	521
D-HCP-1-RO	2.802	687
D-HCP-2-RO	3.652	512
D-HCP-3-RO	1.879	459
B-HCP-LA	1.051	147
D-HCP-LA	0.659	161
B-HCP-OA	2.536	356
D-HCP-OA	2.219	543

Characterisation

Fourier-transform infrared spectroscopy (Tensor II FT-IR Spectrometer, Bruker) was performed on finely ground samples in the range 350-4000 cm^{-1}. Solid-state ^{13}C NMR spectra were collected on a Bruker Avance NEO 500 (Bruker BioSpin) using a 4 mm triple-resonance MAS probe. Samples (15–25 mg) were packed in 4 mm zirconia rotors and measured at 125.78 MHz with a MAS rate of 14 kHz. Porous properties of HCPs were determined by N_2 adsorption isotherms (Tristar II, Micromeritics) measured at 77 K. Prior to measurement, samples were degassed overnight at 120 °C. Surface areas were calculated by the Brunauer-Emmet-Teller (BET) method using the adsorption branch of the isotherm within the relative pressure (P/P_0) range of 0.05-0.2. The total pore volume (V_{tot}) was determined using the N_2 uptake at P/P_0 = 0.97, and the micropore volume (V_{micro}) was calculated via the t-plot method within the P/P_0 range 0.15-0.4. Thermogravimetric analysis (Discovery TGA, TA instruments) was performed using approximately 20 mg of sample. The sample was heated from room temperature under air flow at a rate of 10 °C/min. High resolution X-ray photoelectron spectroscopy (XPS, Nexsa Photoelectron Spectrometer, Thermo Scientific) was recorded using a step size of 0.1 eV and a pass energy of 50 eV. All XPS measurements employed Al-Kα radiation with a spot size of 400 µm. Evaluation of the spectra was carried out using Avantage software (v5.9931, Thermo Fisher Scientific). During peak deconvolution, the full width at half maximum (FWHM) was allowed to vary within a defined range of 0.5-1.5 eV, and the atomic composition was determined from the peak areas using the integrated scaling factor database ALTHERMO1. Elemental analysis (CHNS-O) was performed on a Eurovector EA 3000 analyser using ~2 mg of sample sealed in 4×6 mm tin vials, with weights recorded on a Sartorius ME5 OCE microbalance. Measurements were carried out in triplicate, with combustion/reduction at 1000 °C (1480 °C for O) and 750 °C, respectively, using high-purity helium (99.999%) as carrier gas.

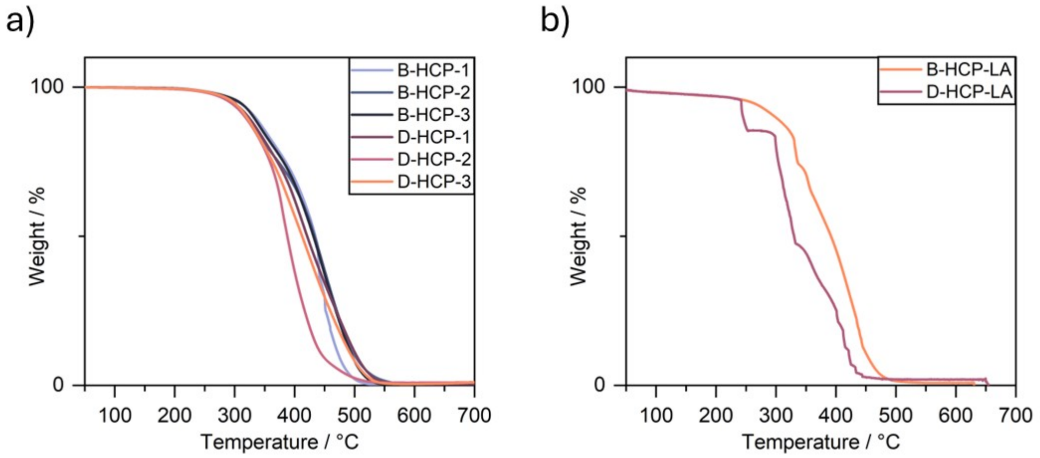

Figure S1. Thermogravimetric analysis of HCPs synthesised using: a) coconut oil b) lauric acid.

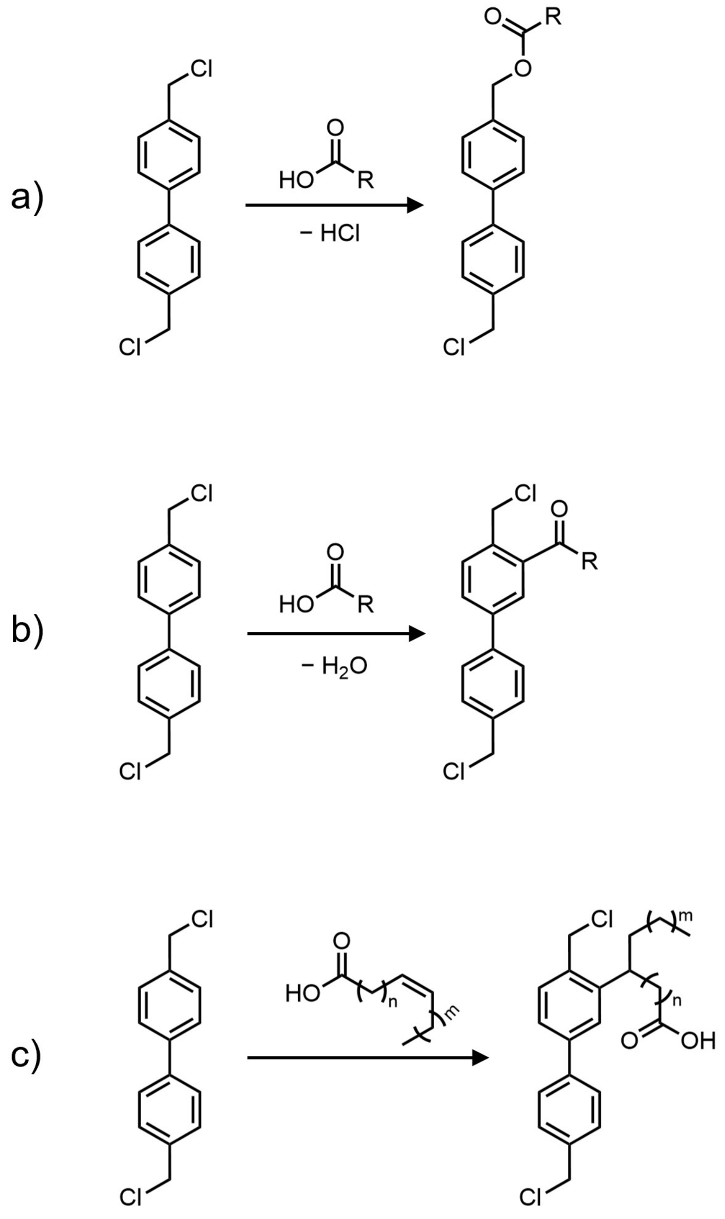

Figure S2. Schematic of the proposed side reactions occurring between the monomer 4,4'-bis(chloromethyl)-1,1'-biphenyl and the fatty acids of the bio-based solvents. a) Nucleophilic attack of the carboxylic acid resulting in ester linkage formation, b) electrophilic aromatic substitution via an acylation-type mechanism, and c) Friedel-Crafts alkylation type reaction via the double bond of unsaturated fatty acids.

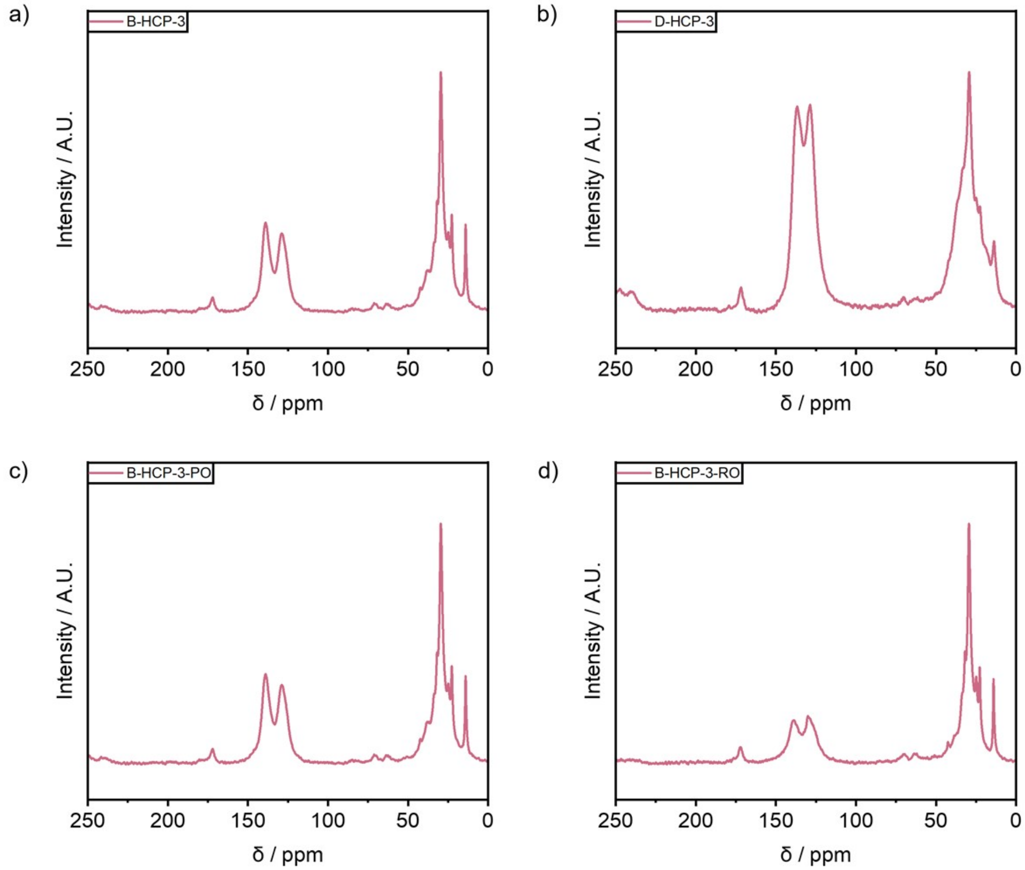

Figure S3. ^{13}C cross-polarisation/magic angle spinning solid-state NMR of a) B-HCP-3, b) D-HCP-3, c) B-HCP-3-PO, and d) B-HCP-3-RO

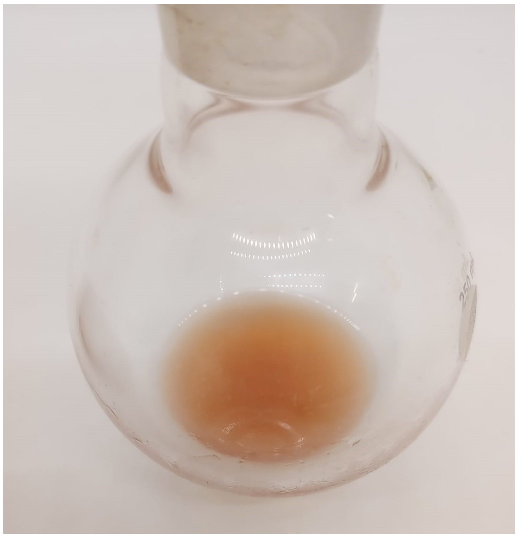

Figure S4. Photograph of coconut oil recovered post-synthesis. The slight orange colour is attributed to trace iron residues.

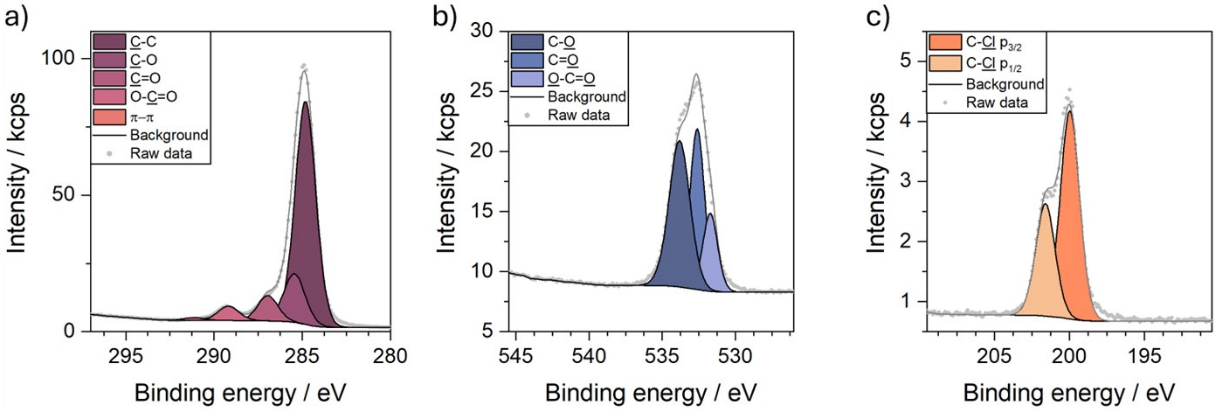

Figure S5. X-ray photoelectron spectroscopy spectra of D-HCP-3: a) High-resolution C 1s spectrum, b) High-resolution O 1s spectrum, and c) High-resolution Cl 2p spectrum.

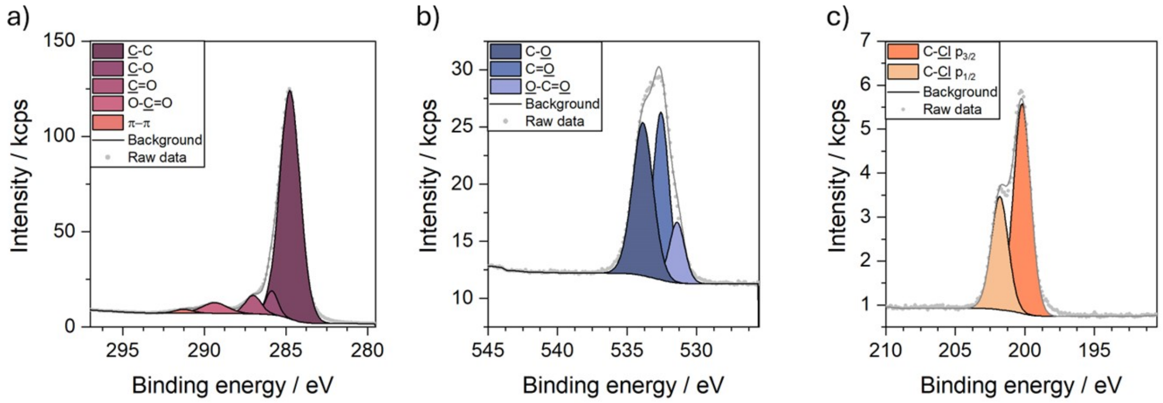

Figure S6. X-ray photoelectron spectroscopy spectra of B-HCP-3-PO: a) High-resolution C 1s spectrum, b) High-resolution O 1s spectrum, and c) High-resolution Cl 2p spectrum.

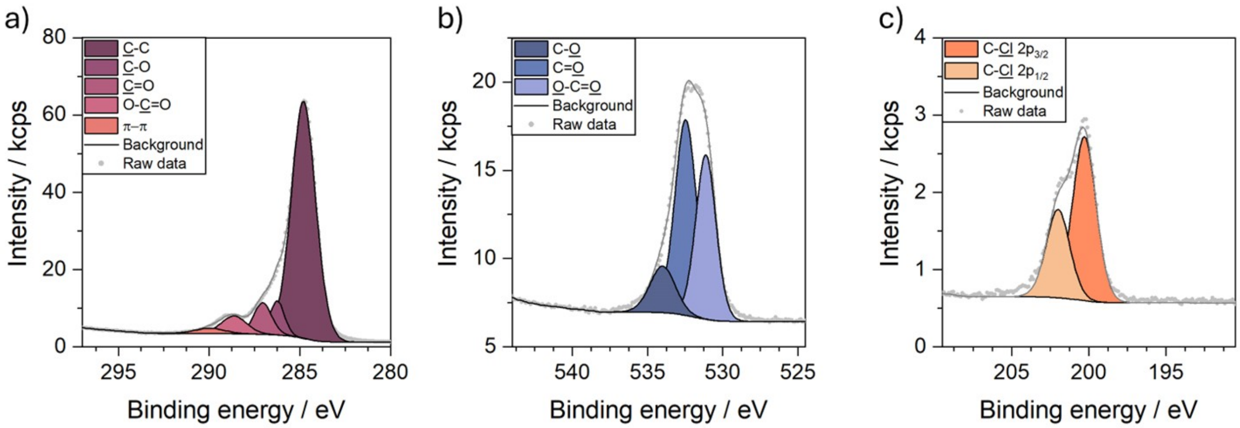

Figure S7. X-ray photoelectron spectroscopy spectra of B-HCP-3-RO: a) High-resolution C 1s spectrum, b) High-resolution S 2p spectrum, and c) High-resolution Cl 2p spectrum.

Table S3. Elemental composition of vegetable oil based HCPs determined by XPS.

	C (wt.%)	N (wt.%)	O (wt.%)	S (wt.%)	Cl (wt.%)
B-HCP-3	86.03 ± 0.25	-	8.48 ± 0.08	-	3.50 ± 0.33
D-HCP-3	84.27 ± 0.01	-	11.06 ± 0.21	-	4.68 ± 0.21
B-HCP-3-PO	82.54 ± 0.15	-	12.76 ± 0.41	-	4.71 ± 0.26
B-HCP-3-RO	81.20 ± 0.13	-	14.41 ± 0.01	-	4.39 ± 0.14

Table S4. Elemental composition of vegetable oil-based HCPs determined by EA.

	C (wt.%)	N (wt.%)	O (wt.%)	S (wt.%)	H (wt.%)
B-HCP-3	81.53 ± 0.04	<0.05	6.74 ± 0.18	0.11 ± 0.01	6.59 ± 0.05
D-HCP-3	78.23 ± 0.07	<0.05	9.07 ± 0.21	0.12 ± 0.01	7.12 ± 0.03
B-HCP-3-PO	74.97 ± 0.20	<0.05	11.61 ± 0.13	<0.02	8.73 ± 0.03
B-HCP-3-RO	75.61 ± 0.86	<0.05	11.46 ± 0.81	<0.02	8.91 ± 0.10

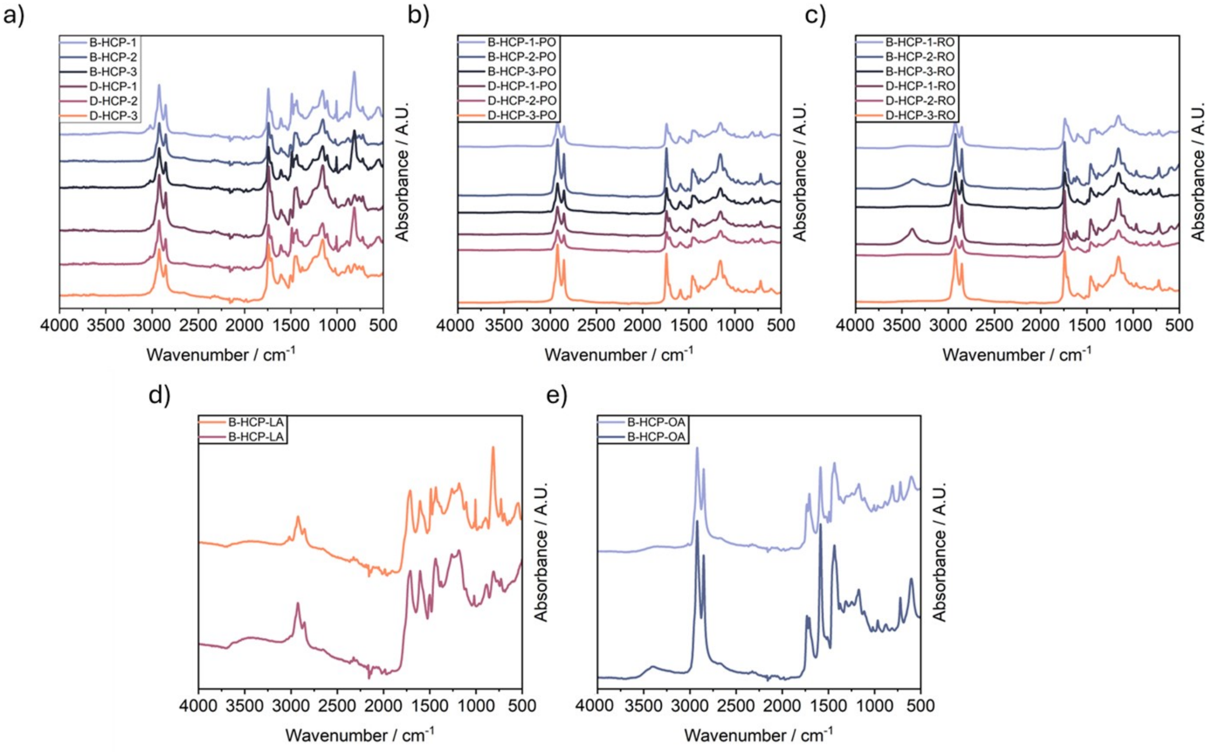

Figure S8. FTIR spectra of HCPs synthesised using: a) coconut oil; b) palm oil; c) rapeseed oil; d) lauric acid; e) oleic acid.

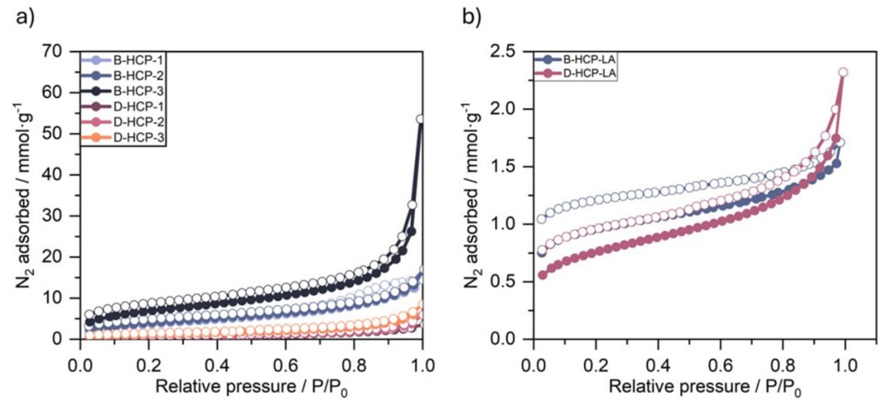

Figure S9. N_2 adsorption-desorption isotherms. Filled symbols represent adsorption and empty symbols represent desorption of HCPs synthesised using: a) coconut oil b) lauric acid**.**

Equation 2. Calculation of complete environmental impact factor (cEF). m is the mass in g and solvents includes both reaction solvent and wash solvents used during workup.

$$cEF = \frac{\sum m(raw\ materials) + \sum m(reagents) + \sum m(solvents) + m(water) - m(products)}{m(product)}$$

Table S5. Masses used in the cEF calculation for HCP synthesised using coconut oil (BHCP-3) and conventional 1,2-dichloethane (BP-HCP) as a solvent.

	BHCP-3	BP-HCP[a]
m (raw materials)	1.0	1.0
m (reagents)	2.0	1.0
m (solvents)	4.6	6.6
m (wash solvents)	250	250
m (water)	0.0	0.0
m (product)	1.0	0.7
**cEF**	**249**	**365**

[a] Referenced from ACS Appl. Mater. Interfaces 2024, 16, 43, 58566–58572 and normalised to 1 g of monomer for ease of comparison

Table S6. Masses used in the cEF calculation for HCP synthesised using coconut oil (BHCP-3) and conventional 1,2-dichloethane (BP-HCP) as a solvent, considering that the wash solvent was reused 10 times.

	BHCP-3	BP-HCP[a]
m (raw materials)	1.0	1.0
m (reagents)	2.0	1.0
m (solvents)	4.6	6.6
m (wash solvents)	25	25
m (water)	0.0	0.0
m (product)	1.0	0.7
**cEF**	**31**	**47**

[a] Referenced from ACS Appl. Mater. Interfaces 2024, 16, 43, 58566–58572 and normalised to 1 g of monomer for ease of comparison

Table S7. Porous properties of HCPs.

Sample	Surface area ($m^2 \cdot g^{-1}$)	V_{micro} ($cm^3 \cdot g^{-1}$)	V_{tot} ($cm^3 \cdot g^{-1}$)
B-HCP-1	315 ± 15	<0.01	0.50 ± 0.09
B-HCP-2	359 ± 87	<0.01	0.51 ± 0.14
B-HCP-3	447 ± 94	0.015 ± 0.003	0.66 ± 0.41
D-HCP-1	54 ± 2	<0.01	0.09 ± 0.01
D-HCP-2	58 ± 7	<0.01	0.09 ± 0.03
D-HCP-3	96 ± 9	<0.01	0.16 ± 0.03
B-HCP-1-PO	-	-	-
B-HCP-2-PO	-	-	-
B-HCP-3-PO	-	-	-
D-HCP-1-PO	-	-	-
D-HCP-2-PO	-	-	-
D-HCP-3-PO	-	-	-
B-HCP-1-RO	-	-	-
B-HCP-2-RO	-	-	-
B-HCP-3-RO	-	-	-
D-HCP-1-RO	-	-	-
D-HCP-2-RO	-	-	-
D-HCP-3-RO	-	-	-
B-HCP-LA	31 ± 1	<0.01	0.04 ± 0.01
D-HCP-LA	37 ± 4	<0.01	0.05 ± 0.01
B-HCP-OA	-	-	-
D-HCP-OA	-	-	-
